# Electrical Characterization and Hydrogen Peroxide Sensing Properties of Gold/Nafion:Polypyrrole/MWCNTs Electrochemical Devices

**DOI:** 10.3390/s130303878

**Published:** 2013-03-19

**Authors:** Graziella Scandurra, Antonella Arena, Carmine Ciofi, Gaetano Saitta

**Affiliations:** Dipartimento di Ingegneria Elettronica, Chimica e Ingegneria Industriale, Università di Messina, Messina I-98166, Italy; E-Mails: gscandurra@unime.it (G.S.); cciofi@unime.it (C.C.); saitta@unime.it (G.S.)

**Keywords:** H_2_O_2_ sensor, MWCNTs dispersions, Nafion:polypyrrole

## Abstract

Electrochemical devices using as substrates copier grade transparency sheets are developed by using ion conducting Nafion: polypyrrole mixtures, deposited between gold bottom electrodes and upper electrodes based on Multi Walled Carbon Nanotubes (MWCNTs). The electrical properties of the Nafion:polypyrrole blends and of the gold/Nafion:polypyrrole/MWCNTs devices are investigated under dry conditions and in deionized water by means of frequency dependent impedance measurements and time domain electrical characterization. According to current-voltage measurements carried out in deionized water, the steady state current forms cycles characterized by redox peaks, the intensity and position of which reversibly change in response to H_2_O_2_, with a lower detection limit in the micromolar range. The sensitivity that is obtained is comparable with that of other electrochemical sensors that however, unlike our devices, require supporting electrolytes.

## 1. Introduction

Hydrogen peroxide (H_2_O_2_) is a chemical miscible with water and able to cross cell membranes, widely employed in many industrial processes including textile and paper bleaching, sterilization, food and pharmaceutical industry. The fast and reliable detection of hydrogen peroxide at concentrations ranging from micromolar to tens of millimolar is therefore of great importance, because it is demonstrated that exposure to H_2_O_2_ at levels ≥50 μM is cytotoxic to a wide range of animals, plants and bacterial cells in culture. In addition, the quantitative determination of the H_2_O_2_ finds increasing biological applications, as hydrogen peroxide is a by-product in a variety of enzymatic reactions. The analytical techniques commonly employed to detect hydrogen peroxide include spectrophotometry [1], cromathography [2], chemiluminescence [3], fluorescence [4], titrimetry [5] and electrochemistry [6]. Compared to the other techniques, electrochemical sensing, being essentially based on the electro-reduction/oxidation of the analyte, allows fast and sensitive detection of H_2_O_2_, with the advantage of requiring simpler experimental set-up, and without the need of expensive instrumentation. As the direct electro-reduction/oxidation of H_2_O_2_ on commercially available bare gold or carbon electrodes requires high overpotential, electrochemical hydrogen peroxide sensors using such kind of electrodes require to be operated over a potential window where interference problems arising from common electroactive species cannot be avoided. Therefore, most of the research activity on electrochemical hydrogen peroxide sensors is currently addressed to the development and characterization of modified electrodes onto the surface of which reduction/oxidation of hydrogen peroxide is achievable by operating at lower voltage, thus improving the sensor's selectivity. According to the way the electrode is modified, the H_2_O_2_ electrochemical sensors can be distinguished in enzymatic and nonenzymatic. The former ones owe their high sensitivity, selectivity and fast response to the presence of redox active enzymes immobilized onto the top of the electrode surface [7]. Their disadvantage is a lack of stability arising from the alteration that the immobilized enzymes, strongly affected by temperature, pH, humidity and exposure to chemicals, may undergo during sensors fabrication, use and storage. To overcome such problems, electrochemical detection of hydrogen peroxide can be achieved by using non-enzymatic electrodes, modified with nanostructured metal oxides [8,9], nanocomposites based on carbon nanotubes [10,11], and conjugated polymers [12,13]. Once the enzymatic or the enzymeless electrodes have been prepared, their response towards hydrogen peroxide is tested in electrolytic solutions, and the sensing performances are usually found to be affected by the nature and concentration of the supporting electrolyte, and by the pH of the solution. Based on previous experience acquired in the field of capacitive humidity sensors consisting of dielectric layer deposited between metal and carbon nanotubes electrodes [14], we develop a solid state electrochemical device consisting of Nafion:polypyrrole ion conducting membranes, interfaced between a gold electrode and a composite electrode based on MWCNTs. Due to the presence of the ion conducting layer, the gold/Nafion:polypyrrole/MWCNTs cell has the advantage, over many other sensors, to be able to detect the presence of H_2_O_2_ in water at concentration in the micromolar range, without the need of any electrolyte. The voltage-current cycles of the gold/Nafion:polypyrrole/MWCNTs devices, measured in water, are characterized by a broad peak that reversibly changes its intensity and position in response to H_2_O_2_. The current peak position and current intensity are found to be linearly related to the H_2_O_2_ content, in the range between 5 μM and 30 μM.

## 2. Experimental Section

### 2.1. Materials

The ion conducting film is deposited from blends prepared by sonication of mixtures containing commercially available Nafion 117 alcoholic dispersions (purchased from Ion Power, New Castle, DE, USA), and 5% aqueous dispersions of doped polypyrrole (provided by Sigma-Aldrich, St. Luois, MO, USA). Nafion is an ion conducting perfluorosulfonate membrane, applied in a variety of electrochemical devices owing to its good chemical and thermal stability. Nafion:polypyrrole ion conducing membranes are obtained after full solvent evaporation, by drop deposition of blends consisting of alcoholic solutions of Nafion, and water dispersions of polypyrrole. According to the literature, the proton conductivity as well as the water uptake of the Nafion:polypyrrole membranes depends on the polypyrrole content [15]. The higher the polypyrrole concentration is, the denser is the membrane consistency, the lower is the ion mobility and the ion exchange capability. Aimed at developing electrochemical devices able to detect the presence of H_2_O_2_ in water, we use blends containing about 5% polypyrrole by weight, as a compromise to ensure a good proton conductivity and a limited water uptake in order to avoid the detachment of the ion conducting membrane from the plastic substrate.

**Figure 1.**
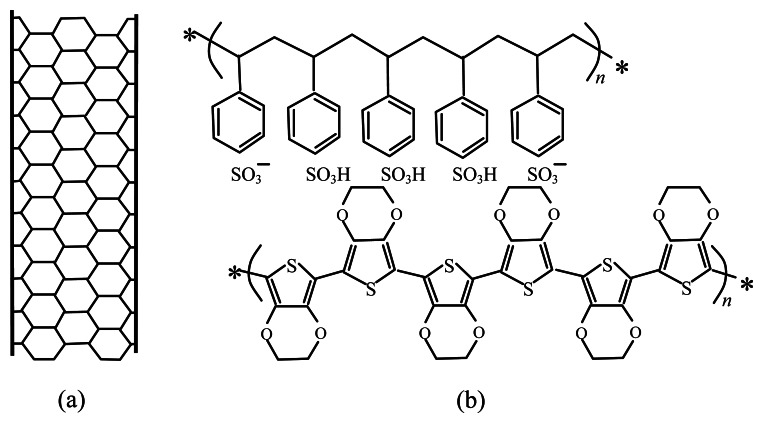
Components of the conducting inks used to develop the top electrode MWCNT (**a**), and PEDOT:PSS (**b**).

The material used as top electrode is a composite consisting of MWCNTs (free sample of ISO HP from Bayer, Leverkusen, Germany), mixed with poly(3,4-ethylenedioxythiophene) poly(styrenesulfonate) (PEDOT:PSS, purchased from Aldrich) (Figure 1). It is well known that unfunctionalised MWCNTs with high length-to-diameter ratio, are not easily dispersible in water or in other common solvents. The simple approach used by us in order to provide stable dispersions of carbon nanotubes, is to mix by ultrasonic treatment the pristine CNTs, and a conjugated polymer (PEDOT) doped with a water-soluble polyelectrolyte (PSS). MWCNTs are wrapped by PEDOT:PSS, forming suspensions with long term stability that likely is ensured by the noncovalent interactions between the delocalized π bond network of carbon nanotubes and the thiophene rings of PEDOT backbone [16].

### 2.2. Design of the Device

**Figure 2.**
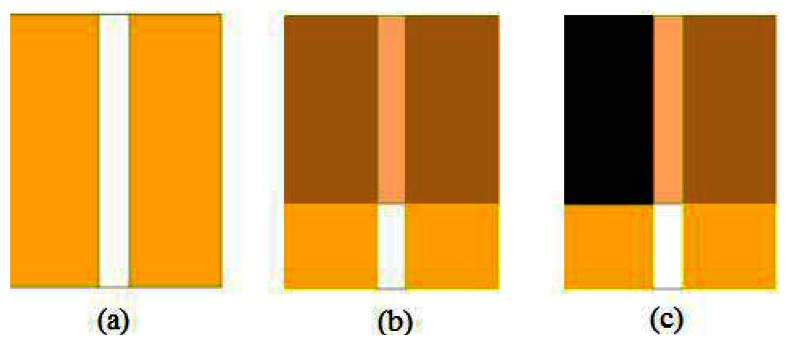
Schematic view of process steps for the realization of the sensors: (**a**) Linearly shaped gold electrodes applied onto the copier grade transparency sheets, developed by thermal evaporation in vacuum; (**b**) deposition of the ion conducting (Nafion:polypyrrole) film; (**c**) deposition of the conducting ink over the Nafion:polypyrrole film, in correspondence of one of the underlying gold electrodes.

The devices consist of an ion conducting film deposited over a couple of linearly shaped bare gold electrodes spaced by about half a mm one from the other (Figure 2). Then, a conducting ink based on multiwalled carbon nanotubes is applied on the ion conducting film, in correspondence of one of the underlying gold electrode. Linearly shaped gold electrodes with an average size of about 4 mm × 8 mm, applied onto the copier grade transparency sheets, are developed by thermal evaporation in vacuum using shadow masks. The devices are accomplished by drop depositing the ion conducting blends over the gold electrodes, and then by depositing a conducting ink over the Nafion:polypyrrole film, in correspondence of one of the underlying gold electrodes. The carbon nanotubes electrodes are applied using an automated dispenser, filled with a stable highly conducting ink based on MWCNT and doped PEDOT.

### 2.3. Materials and Device Characterization

The morphology of the ion conducting Nafion:polypyrrole membranes and of conducting films developed from the MWCNT based ink, is investigated by Scanning Electron Microscopy (SEM) measurements performed using a JEOL 5600 LV electron microscope.

The electrical properties of the gold/Nafion:polypyrrole/MWCNTs are characterized by means of impedance measurements, performed in air by means of an Agilent 4284A LCR meter, in the frequency range between 20 Hz and 1 MHz, using a 100 mV amplitude sinusoidal input. Time domain electrical characterization is performed using the 2400 Keithley sourcemeter, by measuring the current flowing through the gold/Nafion:polypyrrole/MWCNTs devices, in response to triangular voltage inputs. Measurements are carried out at different voltage time rates, over symmetric voltage windows. Sensing tests are performed with the sensors immersed in deionized water. Diluted H_2_O_2_ solutions prepared starting from hydrogen peroxide 35% by weight in water (Aldrich), are injected by using a micro syringe into the solution containing the flexible device under test, in order to increase the H_2_O_2_ concentration in steps of 5 μM, 20 μM, and 50 μM.

## 3. Results and Discussion

**Figure 3.**
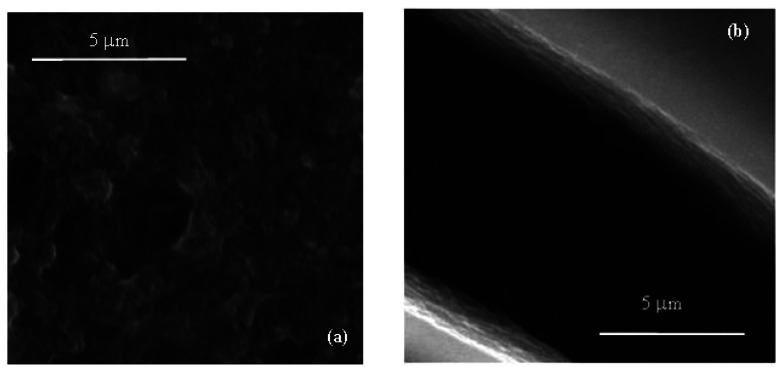
(**a**) Magnified image of a film deposited on silicon from the dispersion of MWCNTs in PEDOT; (**b**) SEM micrograph of Nafion:polypyrrole deposited on a silicon substrate, evidencing the layered structure of the ion conducting film.

**Figure 4.**
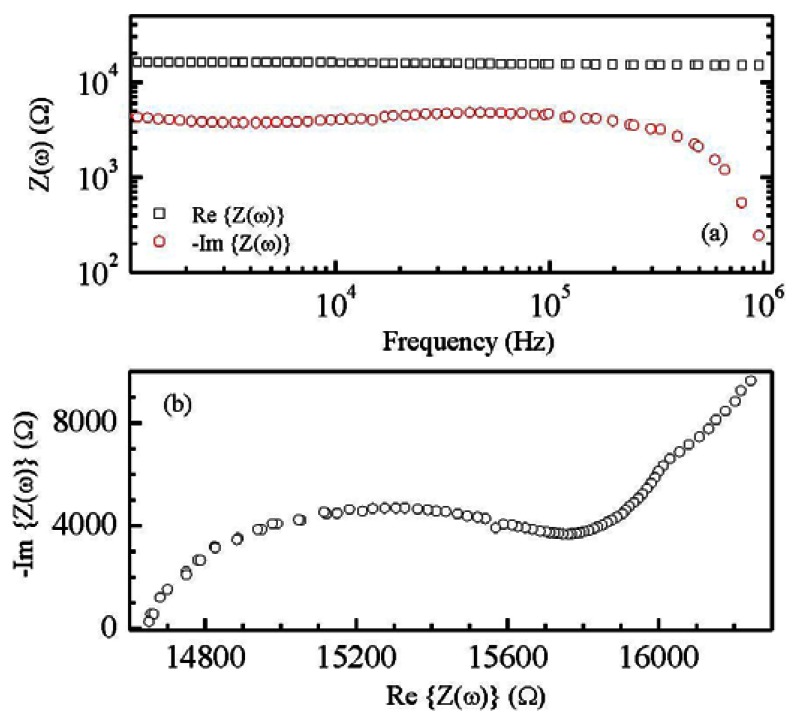
(**a**) Frequency dependence of the complex impedance of a typical Nafion:polypyrrole film; (**b**) Cole Cole plot reporting the opposite of the imaginary part of the impedance versus the real part.

**Figure 5.**
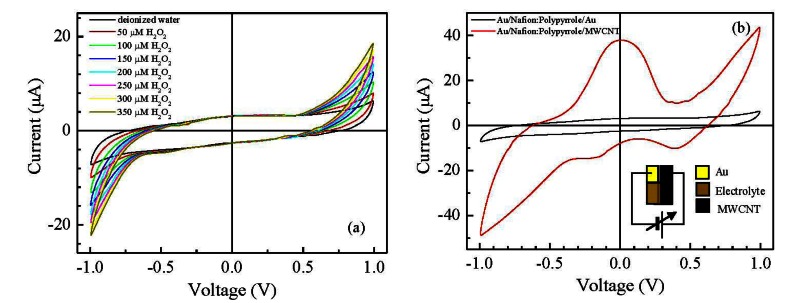
(**a**) Steady state current-voltage cycles of a typical gold/Nafion:polypyrrole/gold device, measured in distilled deionized water (black line), and in the presence of increasing amounts of H_2_O_2_; (**b**) Current-voltage cycle of a typical gold/Nafion:polypyrrole/gold cell, compared to the I–V cycle of a typical gold/Nafion:polypyrrole/MWCNTs cell. Both the curves are measured in steady state, in deionized water. The inset in the right bottom of the figure shows the schematic view of the gold/Nafion:polypyrrole/MWCNTs cell and the voltage polarity.

Films deposited from the conducting inks based on carbon nanotubes have a resistive electrical behavior, with sheet resistance of the order of hundreds Ω per square. According to the SEM micrograph of Figure 3(a) the conducting films have a porous morphology, with MWCNTs well dispersed into the PEDOT:PSS host matrix. Films deposited from the Nafion:polypyrrole blends have smooth surface morphology and seem to be characterized by a layered structure, as it is evidenced by the SEM micrograph of Figure 3(b). From the electrical point of view, the impedance Z(ω) of Nafion:polypyrrole is found to strongly depend on the environmental humidity and temperature. Measurements shown in Figure 4(a), performed at 60% RH and at 25 °C on a typical film deposited between symmetric gold electrodes, reveal a resistive-capacitive behavior, with the real part of Z nearly constant over the investigated frequency range, and the opposite of the imaginary part that decreases with frequency above 10^5^ Hz. The opposite of the imaginary part of the impedance, plotted against the real part in Figure 4(b), forms a broad flat semicircle, corresponding to the high frequency region, that according to the literature [17], evidences the occurrence of processes at the interface between the electrode and the electrolyte. The quasi linear trend observed in the rightmost side of the plot of Figure 4(b), suggests that the response to low frequency electrical inputs is dominated by the diffusion of counterions through the Nafion:polypyrrole film [17]. Figure 5 shows the typical steady state current-voltage plot of a Nafion:polypyrrole film, deposited onto symmetric gold electrodes. Measurements are performed in deionized water by cycling the voltage between −1 V and +1 V, at a voltage change rate of 50 mV/s, before and after the subsequent addition of hydrogen peroxide in steps of 50 μM. It can be noticed that the current forms a closed loop. Such a behavior may be, as it is in the case of double-layer capacitor, the effect of subsequent charging and discharging of the double layer that forms at the interfaces between the electrodes and the ion conducting material. The I–V cycles of Figure 5a have symmetrical shape, and do not exhibit any current peak. While the current intensity measured over a voltage window between −0.5 V and 0.5 V seems to be rather insensitive to the presence of H_2_O_2_, the magnitude of the forward and reverse currents outside such a voltage range is found to increase as the H_2_O_2_ concentration increases. This type of response to H_2_O_2_ is ascribable to the polypyrrole film [18].

**Figure 6.**
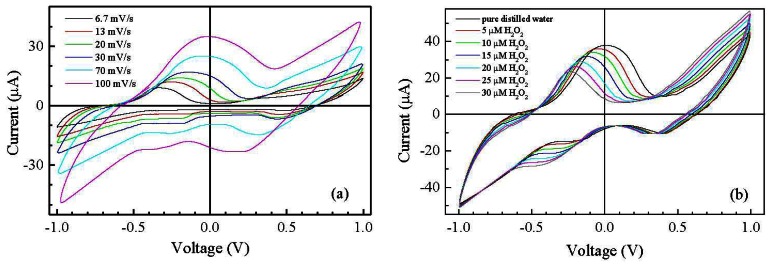
(**a**) Steady state current-voltage cycles of a typical gold/Nafion:polypyrrole/MWCNTs, measured over the same voltage range in pure deionized water, evidencing the shift of the current peaks as a function of the voltage change rate; (**b**) Current-voltage cycle of a typical gold/Nafion:polypyrrole/MWCNTs device, measured in distilled deionized water (black line), and in the presence of increasing amounts of H_2_O_2_. Scan speed is 50 mV/s.

In order to improve the sensitivity towards hydrogen peroxide, the symmetric gold/Nafion:polypyrrole/gold devices are modified by applying a conducting ink based on MWCNTs [19], on top of the ion conducting layer, in correspondence of one of the underlying gold electrodes. The presence of MWCNT dispersed in PEDOT:PSS has significant effects on the current intensity and on the shape of the current-voltage cycles. Compared to that of Nafion:polypyrrole deposited on symmetric gold electrodes, the steady state current-voltage cycle of the typical gold/Nafion:polypyrrole/MWCNTs cell [shown in Figure 5(b)] encloses a larger area. In addition, the current-voltage cycles are characterized by the appearance of a broad forward current peak, centered at about 100 mV, and by a couple of reverse current peaks, positioned at 390 mV and −220 mV, respectively. The enlargement of the steady state current loop and the appearance of current peaks after the application of the MWCNTs ink on top of the Nafion:polypyrrole film can be explained in terms of the interaction between the carbon nanotubes and the conjugated polymer. In particular, the increased current can be the effect of the increased capacitance ascribable to the high surface interfaces between the porous carbon nanotubes film and the conjugated polymer. In addition, as it is discussed by Wanekaya *et al.* [20] in their paper concerning polypyrrole films doped with functionalized Single-Walled Nanotubes (SWNTs), the presence of highly conducting carbon nanotubes in polypyrrole favors the electron transfer of redox processes and weakens the electrokinetic polarization. The forward and reverse current peaks of Figure 5(b) can be therefore ascribed to faradaic processes involving polypyrrole at the carbon nanotubes electrode. The separation between the forward and reverse current peaks suggests that the related faradaic processes are not-reversible. As a further evidence of this, the results of current-voltage measurements performed at different scanning speeds in pure deionized water, shown in Figure 6(a), reveal that the position of the current peaks changes with the time rate of the voltage change. The shifts of the current peaks with the scan rate, evidence that the current response is delayed with respect to the applied voltage input, because the kinetic of the electrochemical processes is slow compared to the voltage scan rate. Figure 6(b) compares the steady state current-voltage cycles measured at the scan rate of 50 mV/s on a typical gold/Nafion:polypyrrole/MWCNTs device immersed in water, before and after the addition of calibrated amounts of hydrogen peroxide.

**Figure 7.**
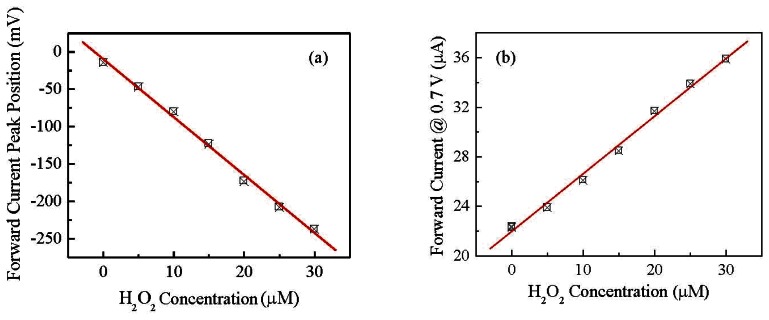
(**a**) Forward current peak position of a gold/Nafion:polypyrrole/MWCNTs device and (**b**) its forward current intensity measured at 0.7 V as a function of H_2_O_2_ concentration. Data are extracted from the results of the measurements shown in Figure 5(b).

The results clearly show that position and intensity of the forward and reverse current peaks are significantly affected by the presence of H_2_O_2_. In particular, it can be observed that while the forward current peak shifts towards negative voltages and decreases, the reverse peak, initially positioned at about −220 mV, moves towards more negative voltages and grows. The response towards hydrogen peroxide is found to be reversible: the shape of the current-voltage cycles as well as the peaks' position and intensity are restored to their initial state after rinsing the device in copious amount of deionized water. Figure 7(a) shows how the forward current peak position changes in response to increasing concentrations of H_2_O_2_.

**Figure 8.**
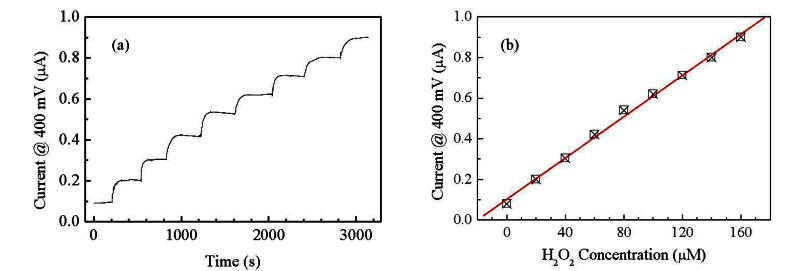
(**a**) Time evolution of the current measured at constant voltage on a gold/Nafion:polypyrrole/MWCNT cell in deionized water, in response to H_2_O_2_ subsequent injections in steps of 20 μM; (**b**) plateau values of the current as function of the hydrogen peroxide concentration.

Since both the peaks positions and their amplitudes are a function of the H_2_O_2_ concentration, we may want to avoid the regions where the peaks occur for defining a simpler electrical output for the sensor, even if this may result in a lower sensitivity. For instance we can select the forward current measured at 0.7 V as the output of the sensor, well outside the region where the peaks occur. The possibility of using the gold/Nafion:polypyrrole/MWCNTs as hydrogen peroxide sensor, is confirmed by the results of measurements, shown in Figure 8(a), of the d.c. current measured at constant voltage in deionized water, in response to subsequent injections of the analyte. It can be noticed that in correspondence to each injection step, the current undergoes a transient phase of increase, and then reaches a plateau. As it is shown in Figure 8(b), the plateau values of the current are found to increase linearly with the hydrogen peroxide concentration.

According to the experimental results, the gold/Nafion:polypyrrole/MWCNTs cell is a resistive-capacitive system. Its current-voltage cycles measured in water, are characterized by forward and reverse current peaks, indicating that the capacitance of the device arises also from faradaic processes. In the presence of hydrogen peroxide, the redox peaks ascribable to electrochemical processes at the MWCNT-Pedot electrode, modify both their position and intensity. Although a complete understanding of the sensing mechanism would require more detailed investigation, the reversible changes observed in the current-voltage plots may be related to the remarkable role that the composite MWCNT/Pedot electrodes have on the elctrocatalytic reduction of H_2_O_2_, in terms of low overvoltage and high reduction current [19].

Compared to many other H_2_O_2_ electrochemical sensors reported in the literature, our cell has the advantage to be able to detect the presence of hydrogen peroxide without the need of any supporting electrolyte. As far as the sensing performances are concerned, the sensitivity S estimated from the slope of the linear calibration curve in Figure 8(b) is S = 1.47 μA·μM^−1^·cm^−2^. Table 1 compares the sensitivity of our sensor with the sensitivity other sensors reported in literature [21–24]. In judging the performances of our sensor in terms of sensitivity, it must be noted that all other reported data refers to measurements in the presence of a support electrolyte: no electrolyte is present in our case. An estimation of the response time of the sensor can be obtained by the chronoamperometric measurements reported in Figure 8(a). The average time required for the current to change from its previous value to the 90% of its final value is about 80 s.

## 4. Conclusions

Flexible devices deposited on copier grade transparency sheets, based on Nafion blended with polypyrrole, and having gold and carbon nanotubes electrodes, have been prepared and electrically characterized in the frequency and time domain. The steady state current-voltage characteristics measured in deionized water, have a cyclic shape dominated by forward and reverse current peaks. The shape of the cycles, the current peaks positions, and the current intensity, are found to reversibly respond to the presence of H_2_O_2_. In particular, the forward current intensity measured at 0.7 V, and the forward current peak position, are found to be linearly related to hydrogen peroxide concentration in the range between 5 μM and 30 μM. Preliminary results of chronoamperometric measurements reveal that the current measured at 0.4 V linearly grows with H_2_O_2_ concentration between 20 μM and 160 μM. These results are quite encouraging in view of developing simple and inexpensive disposable hydrogen peroxide sensors. Future work will be focused on devising viable measurement approaches for exploiting the dynamic response of the sensor, where a higher sensitivity can be obtained with respect to the DC response, and on the investigation of interference effects on both the DC and dynamic response.

## Figures and Tables

**Table 1.** Sensitivity of our sensor compared to that of other electrochemical sensors.

Sensor	Sensitivity (μA·μM^−1^·cm^−2^)	Ref.
Gold/Nafion:polypyrrole/MWCNTs	1.47	This work
CG electrode modified with FeNiPt nanoparticles	2.45	[21]
Gold electrode modified with Cu_2_O nanowires and Nafion	7.45	[22]
CG electrode modified with SnO_2_-supported Pt nanoparticles	2.411	[23]
Gold electrode modified with Prussian blue nanorods	3.00	[24]
